# Influenza A(H1N1)pdm09 Virus Infection in a Captive Giant Panda, Hong Kong

**DOI:** 10.3201/eid2512.191143

**Published:** 2019-12

**Authors:** Paolo Martelli, Jade L.L. Teng, Foo-Khong Lee, Kai-Yan Yeong, Jordan Y.H. Fong, Suk-Wai Hui, Kwok-Hung Chan, Susanna K.P. Lau, Patrick C.Y. Woo

**Affiliations:** Ocean Park Corporation, Hong Kong, China (P. Martelli, F.-K. Lee, S.-W. Hui);; The University of Hong Kong, Hong Kong (J.L.L. Teng, K.-Y. Yeong, J.Y.H. Fong, K.-H. Chan, S.K.P. Lau, P.C.Y. Woo);; State Key Laboratory of Emerging Infectious Diseases at the University of Hong Kong, Hong Kong (J.L.L. Teng, K.-H. Chan, S.K.P. Lau, P.C.Y. Woo);; Collaborative Innovation Center for Diagnosis and Treatment of Infectious Diseases at the University of Hong Kong, Hong Kong (S.K.P. Lau, P.C.Y. Woo)

**Keywords:** influenza A(H1N1)pdm09, giant panda, influenza, Hong Kong, viruses, respiratory infections

## Abstract

We report influenza A(H1N1)pdm09 virus infection in a captive giant panda in Hong Kong. The viral load peaked on day 1 and became undetectable on day 5, and an antibody response developed. Genome analysis showed 99.3%–99.9% nucleotide identity between the virus and influenza A(H1N1)pdm09 virus circulating in Hong Kong.

Since 2009, influenza A(H1N1)pdm09 virus (pH1N1) has been circulating seasonally worldwide and causing substantial illness, hospitalization, and death in humans every year. The virus has also caused infection in mammals and birds in addition to humans (*1**–**3*).

The giant panda (*Ailuropoda melanoleuca*) is considered a National Treasure of China with the highest legal protection and dedicated recovery programs. Any emerging infection in giant pandas is of utmost importance because they may not have adequate immunity against the pathogen, implying that such infection may rapidly spread to other giant pandas, leading to large outbreaks and fatalities (*4*). In this article, we describe a case of pH1N1 infection in a captive giant panda in an oceanarium in Hong Kong, China.

## The Study

Ocean Park Hong Kong is a financially independent not-for-profit zoological park, oceanarium, and amusement park housing >5,000 marine and terrestrial animals of >500 species. There are 2 buildings for giant pandas in the park; 1 houses a 32-year-old male giant panda and the other a breeding pair.

On November 14, 2018, the 13-year-old male panda of the breeding pair was lethargic and had low appetite. Examination showed yellowish-brown mucoid nasal discharge, tachypnea (respiratory rate >60 breaths/min), and abdominal breathing. On day 2, his condition worsened, and he showed little appetite, persistent nasal discharge, and cough. Attempts at rectal temperature measurement and blood collection were unsuccessful in the first 2 days. We initiated treatment with ciprofloxacin, carprofen, bromhexine, and β-glucan and fogged his living quarters twice daily with F10 antiseptic solution (1:250 dilution) containing benzalkonium chloride and polyhexanide. Clinical surveillance performed on staff members of the park at the time when the giant panda was ill revealed that none of the animal caretakers had influenza-like illness around that time. Additional measures included placing rat traps to test resident rodents for influenza and increased biosecurity to limit contact between the breeding pair and between the staff and giant pandas at both panda facilities.

On day 3, the panda’s conditions and appetite improved. Nasal discharge was unchanged, but he only coughed occasionally. Rectal temperature was normal. Blood examination revealed leukocytosis with marked neutrophilia and lymphopenia, hypoferremia, and increased fibrinogen and globulins. He gradually improved in the next 5 days and has remained asymptomatic for 9 months after the onset of illness.

On day 1 of his illness, we collected nasal swab specimens for virologic studies; we collected additional nasal samples on days 2, 3, and 5 for viral load measurement. We took serial serum samples before and after the illness for serologic studies. Veterinary surgeons performed all sample collection. 

We performed rapid antigen detection using BinaxNOW Influenza A & B Card (Alere, https://www.alere.com) and determined viral loads by quantitative real-time reverse transcription PCR (RT-PCR) targeting the M gene (*5*). We performed cell culture using MDCK cells inoculated with the first nasal swab sample; we examined it for cytopathic effect at 72 h. We performed serologic analyses using hemagglutination inhibition (HI) and microneutralization (MN) assays (*6*,*7*). We determined the complete genome sequencing of the culture isolate by Illumina HiSeq1500 (https://www.illumina.com) as described previously (*8*,*9*). We deposited the genome sequence in the GISAID database (http://platform.gisaid.org; accession no. EPI1493152 and nos. EPI1493160–6).

**Figure 1:**
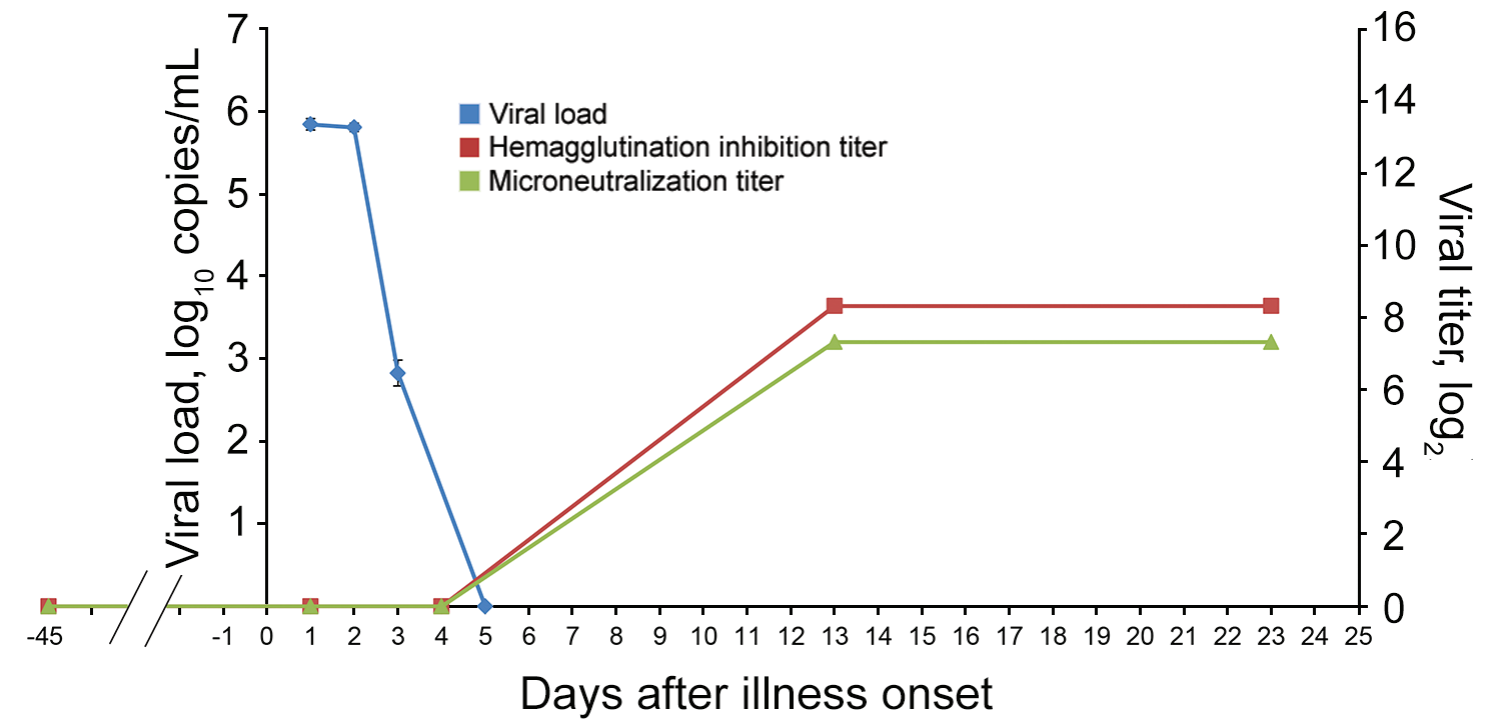
Viral load and serologic response to influenza A(H1N1)pdm09 in nasal and serum samples from an infected giant panda in Hong Kong, China. Hemagglutination inhibition (red) and microneutralization (green) antibody titers are shown on a log_2_ scale, and viral load (blue) shown as mean viral load ± SD (log_10_ M gene copies/mL).

Rapid antigen detection on the nasal swab specimen collected on day 1 was positive for influenza A virus. The viral load (± SD) in the nasal swabs on day 1 of the illness was 5.84 ± 0.07 log_10_ copies/mL; on day 2, 5.81 ± 0.05 log_10_ copies/mL; and on day 3, 2.83 ± 0.16 log_10_ copies/mL. On day 5, viral loads became undetectable (Figure 1). MDCK cells inoculated with the nasal sample showed cytopathic effect on day 3 of incubation with cell rounding, progressive degeneration, and detachment. Serologically, HI and MN antibodies against pH1N1 were undetectable >40 days before the onset of the illness and on days 1 and 4 of the illness, but high titers (HI, 1:320; MN, 1:160) were detected in the second and the fourth week after the onset of the illness (Figure 1). The other 2 giant pandas did not develop any clinical signs, and their nasal swab specimens remained negative by RT-PCR.

**Figure 2:**
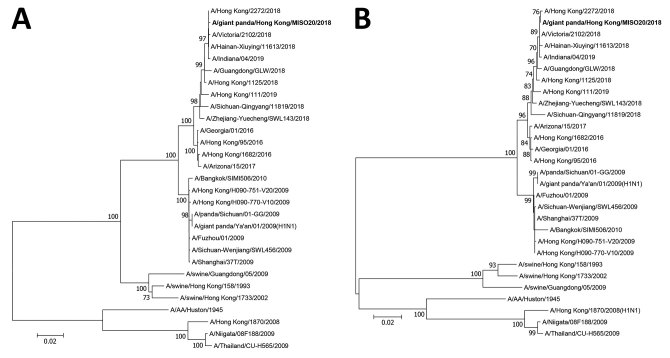
Phylogenetic analyses of (A) hemagglutinin and (B) neuraminidase gene sequences of influenza A(H1N1)pdm09 (A/giant panda/Hong Kong/MISO20/2018) isolated from a giant panda in Hong Kong, China (bold), and other previously characterized strains retrieved from GISAID. The trees were constructed by the neighbor-joining method using Kimura 2-parameter in MEGA6 (http://www.megasoftware.net). A total of 1,691 nt positions in hemagglutinin and 1,404 in neuraminidase genes were included in the analyses. Bootstrapping was performed with 1,000 replicates; only bootstrap values >700 are shown. Scale bars indicate nucleotide substitutions per site.

Whole-genome sequence analysis showed that the influenza virus we isolated from the giant panda (A/giant panda/Hong Kong/MISO20/2018) was closely related to other pH1N1 viruses circulating among humans in 2018, sharing 99.3%–99.9% nucleotide identities (Table). Phylogenetic analyses based on the hemagglutinin (HA) and neuraminidase (NA) gene sequences showed that A/giant panda/Hong Kong/MISO20/2018 was most closely related to the human pH1N1 strain A/Hong Kong/2272/2018, which was circulating in Hong Kong at the time at which the giant panda acquired the infection (Figure 2; Appendix Figure). There were only 2 bases difference between the HA genes and 4 bases difference between the NA genes of A/giant panda/Hong Kong/MISO20/2018 and A/Hong Kong/2272/2018, but 60 bases difference between the HA genes and 45 bases difference between the NA genes of A/giant panda/Hong Kong/MISO20/2018 and A/giant panda/01/Ya’an/2009, a pH1N1 virus previously isolated in giant panda in China (*1*). Phylogenetic analyses based on the other gene segments displayed similar topologies (Appendix Figure). Detailed annotation of the genome sequence of the giant panda isolate revealed features essential for transmission and replication of pH1N1 in other mammalian species. For example, A/giant panda/Hong Kong/MISO20/2018 also possessed glutamine at position 226 (H3 numbering) in HA and alanine at position 271 in polymerase protein 2 (*1*).

**Table:** Comparison of influenza A(H1N1)pdm09 isolated from a giant panda (A/giant panda/Hong Kong/MISO20/2018) with other representative H1N1 subtype isolates by gene segment*

Isolate	Nucleotide identity, %							
	PB2	PB1	PA	HA	NP	NA	M	NS
A/Hong Kong/2272/2018	99.90	99.90	99.90	99.80	99.80	99.70	99.80	99.70
A/Hainan-Xiuying/11613/2018	99.90	99.70	99.90	99.80	99.70	99.70	99.70	99.80
A/Victoria/2102/2018	99.80	99.60	99.80	99.70	99.60	99.90	99.60	99.60
A/Hong Kong/1125/2018	99.30	99.50	99.50	99.40	99.50	99.50	99.60	99.70
A/Guangdong/GLW/2018	98.90	99.50	99.60	99.00	99.40	99.50	99.30	99.70
A/Zhejiang-Yuecheng/SWL143/2018	99.10	99.20	99.40	98.80	99.30	99.10	99.20	99.50
A/Sichuan-Qingyang/11819/2018	99.00	99.10	99.00	98.50	99.00	98.30	99.20	99.60
A/Hong Kong/111/2019	99.00	98.90	99.40	98.70	99.00	99.00	99.20	99.30
A/Indiana/04/2019	99.90	99.70	99.80	99.70	99.70	99.70	99.70	99.80
A/Arizona/15/2017	98.80	99.00	99.10	98.10	98.90	98.70	99.00	99.20
A/Georgia/01/2016	99.00	99.10	99.20	98.40	99.10	98.70	99.10	99.50
A/Hong Kong/1682/2016	98.90	99.10	99.00	98.20	99.10	98.70	99.00	99.60
A/Hong Kong/95/2016	99.00	99.10	99.30	98.40	99.00	98.70	99.10	99.50
A/Bangkok/SIMI506/2010	97.00	97.30	97.70	96.40	97.50	96.00	97.80	96.80
A/Hong Kong/H090–751-V20/2009	97.30	97.60	97.60	96.60	97.70	96.90	98.10	96.90
A/Shanghai/37T/2009	97.30	97.60	97.70	96.60	97.70	97.00	98.10	96.70
A/Fuzhou/01/2009	97.40	97.60	97.70	96.60	97.70	97.00	98.10	92.60
A/Sichuan-Wenjiang/SWL456/2009	97.40	97.70	97.70	96.60	97.70	96.90	98.10	96.90
A/Hong Kong/H090–770-V10/2009	97.30	97.70	97.70	96.50	97.60	97.00	98.10	96.90
A/giant panda/Ya'an/01/2009	97.30	97.50	97.50	96.40	97.50	96.70	98.10	96.80
A/panda/Sichuan/01-GG/2009	97.30	97.50	97.50	96.40	97.50	96.70	98.10	96.80
A/Niigata/08F188/2009	82.80	80.10	82.50	76.20	83.30	78.00	86.70	80.50
A/Thailand/CU-H565/2009	82.70	80.00	82.60	75.90	83.30	77.90	86.60	81.00
A/swine/Guangdong/05/2009	82.20	79.60	81.60	88.90	92.10	79.30	86.80	91.30
A/Hong Kong/1870/2008	82.70	80.60	82.70	76.00	83.60	77.70	87.00	81.70
A/swine/Hong Kong/1733/2002	81.70	79.30	81.30	89.20	92.60	79.50	86.00	90.40
A/swine/Hong Kong/158/1993	82.10	79.70	82.00	90.20	93.50	79.30	87.20	96.70
A/AA/Huston/1945	84.80	81.80	83.40	78.30	85.10	80.20	88.90	83.60

*HA, hemagglutinin; M, matrix; NA, neuraminidase; NP, nucleoprotein; NSP, nonstructural protein; PA, polymerase; PB1, polymerase basic 1; PB2, polymerase basic 2.

## Conclusions

We documented a case of influenza infection caused by pH1N1 virus in a captive giant panda in Hong Kong. The viral load was >6 × 10^5^ copies/mL during the first 2 days of the illness and decreased to an undetectable level on day 5. The decrease in viral load was coupled with development of antibody response. Complete genome sequencing and phylogenetic analysis showed that the pH1N1 virus from the giant panda differed from the influenza virus circulating in Hong Kong at that time by only 2–24 bases. In 2014, pH1N1 infection was reported in giant pandas at the Conservation and Research Center for the Giant Panda in Sichuan (*1*). That pH1N1 virus, A/giant panda/01/Ya’an/2009, was also closely related to the pH1N1 strains circulating in humans during 2009 (*1*). 

These findings show that influenza A virus infection in this giant panda was not an isolated case and that these infections have happened not only in mainland China. Our findings indicate that the influenza virus in giant pandas was most likely directly or indirectly from humans with seasonal influenza. Of interest, respiratory infection in a sloth bear due to pH1N1 has also been observed in a zoo in the United States in 2014, indicating that pH1N1 can probably infect a variety of bears (*2*).

In animal species with no preexisting immunity against an infectious agent, a new intrusion of the pathogen may result in high fatalities. Transmission of a new strain of influenza from birds and poultry to humans has resulted in many epidemics (*10**–**15*). Because the inactivated vaccine against pH1N1 has been widely used in humans and is effective in mice, pigs, and ferrets, it might be worthwhile to test its immunogenicity in giant pandas. Moreover, caretakers working at these parks who are infected with influenza, even with mild illness or in recovery, should not work near the animals.

Appendix Additional information about influenza A(H1N1)pdm09 in a giant panda, Hong Kong.
