# Supplementary material for: Influenza A(H1N1)pdm09 Virus Infection in a Captive Giant Panda, Hong Kong
Source: Emerg Infect Dis. 2019 Dec;25(12):2303–6. doi: 10.3201/eid2512.191143 (PMC6874238; doi:10.3201/eid2512.191143)
Supplement: Appendix — Additional information about influenza A(H1N1)pdm09 in a giant panda, Hong Kong. [file 19-1143-Techapp-s1.pdf]

Article DOI: https://doi.org/10.3201/eid2512.191143

Influenza A(H1N1)pdm09 Virus Infection in a Captive Giant Panda, Hong Kong

Appendix

A

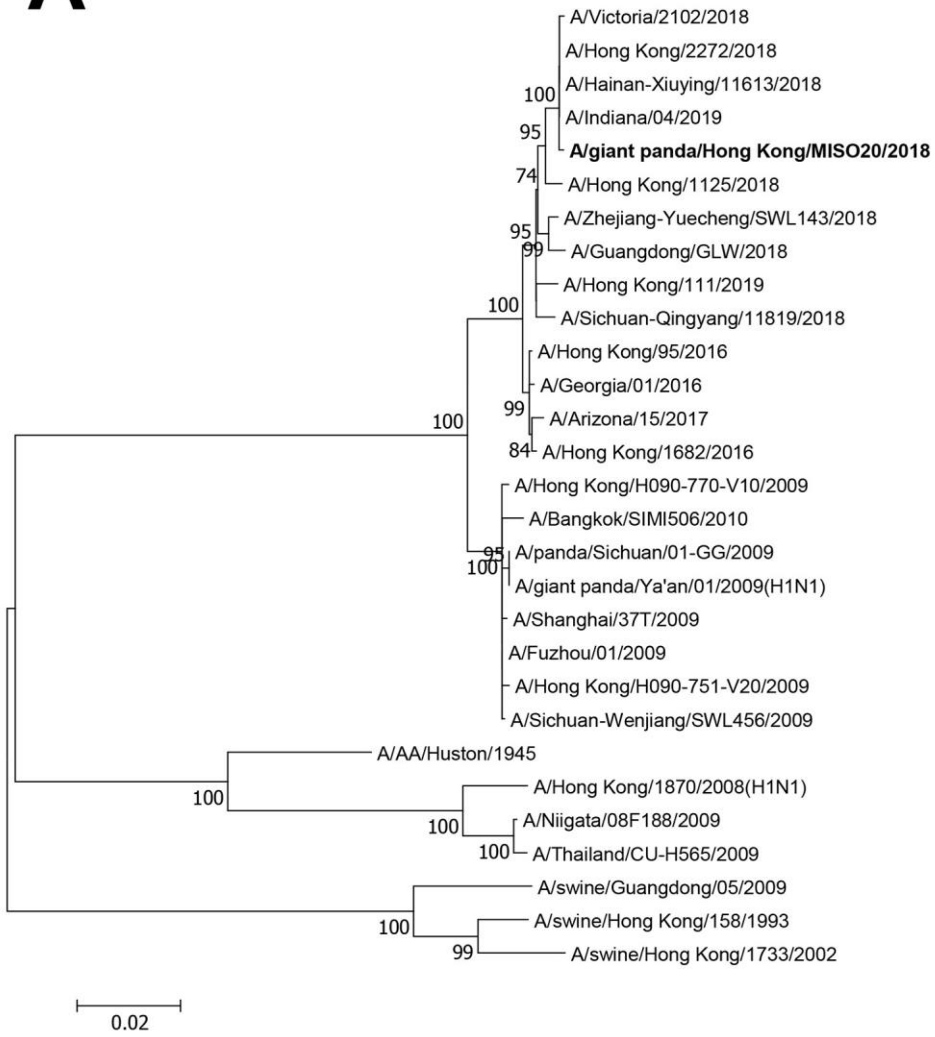

B

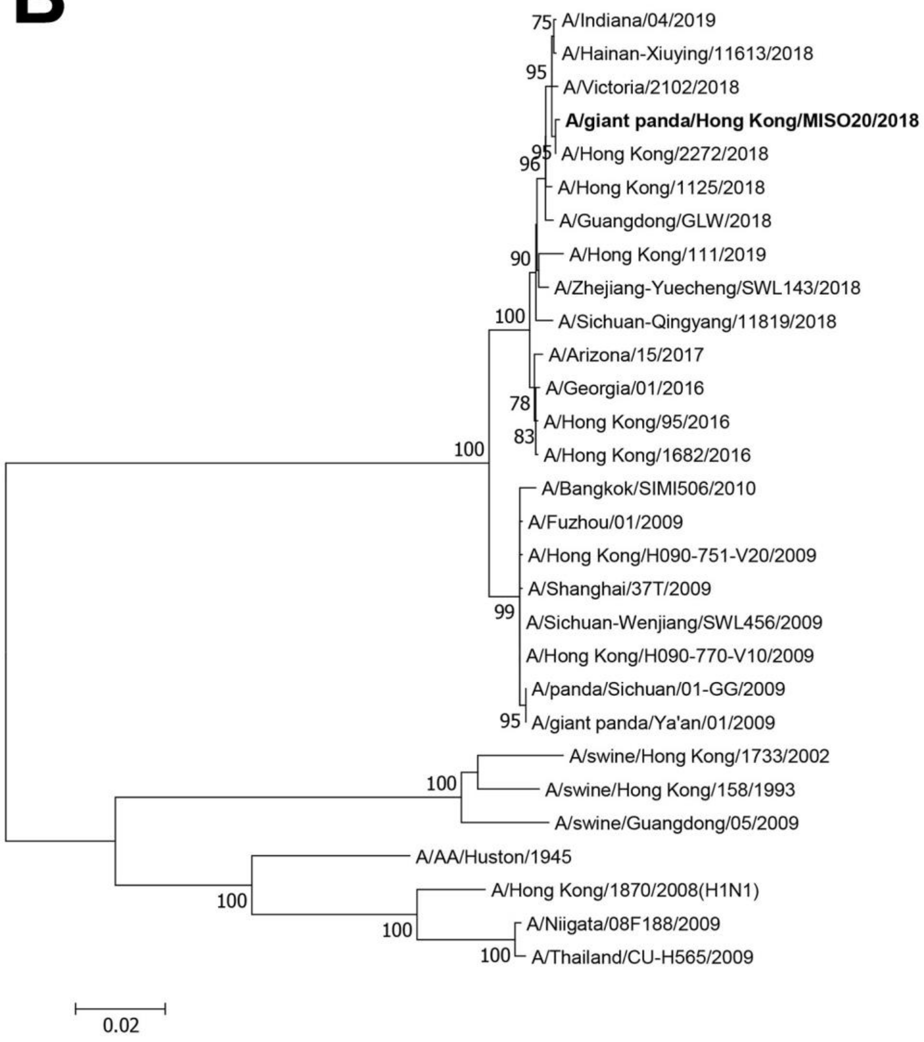
A/Indiana/04/2019
A/Hainan-Xiuying/11613/2018
A/Victoria/2102/2018
A/giant panda/Hong Kong/MISO20/2018
A/Hong Kong/2272/2018
A/Hong Kong/1125/2018
A/Guangdong/GLW/2018
A/Hong Kong/111/2019
A/Zhejiang-Yuecheng/SWL143/2018
A/Sichuan-Qingyang/11819/2018
A/Arizona/15/2017
A/Georgia/01/2016
A/Hong Kong/95/2016
A/Hong Kong/1682/2016
A/Bangkok/SIMI506/2010
A/Fuzhou/01/2009
A/Hong Kong/H090-751-V20/2009
A/Shanghai/37T/2009
A/Sichuan-Wenjiang/SWL456/2009
A/Hong Kong/H090-770-V10/2009
A/panda/Sichuan/01-GG/2009
A/giant panda/Ya'an/01/2009
A/swine/Hong Kong/1733/2002
A/swine/Hong Kong/158/1993
A/swine/Guangdong/05/2009
A/AA/Huston/1945
A/Hong Kong/1870/2008(H1N1)
A/Niigata/08F188/2009
A/Thailand/CU-H565/2009
75
95
95
96
90
100
78
83
100
99
95
100
100
100
100
0.02

C

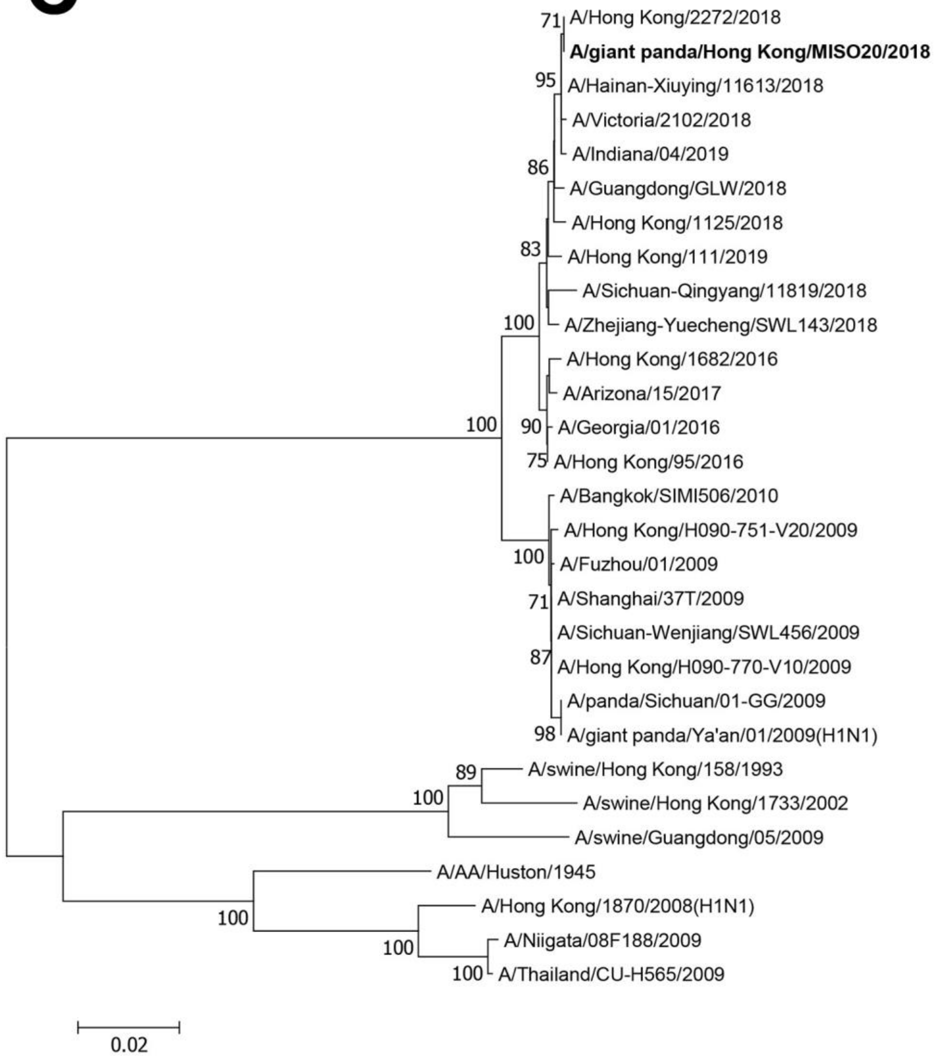
A/Hong Kong/2272/2018
A/giant panda/Hong Kong/MISO20/2018
A/Hainan-Xiuying/11613/2018
A/Victoria/2102/2018
A/Indiana/04/2019
A/Guangdong/GLW/2018
A/Hong Kong/1125/2018
A/Hong Kong/111/2019
A/Sichuan-Qingyang/11819/2018
A/Zhejiang-Yuecheng/SWL143/2018
A/Hong Kong/1682/2016
A/Arizona/15/2017
A/Georgia/01/2016
A/Hong Kong/95/2016
A/Bangkok/SIMI506/2010
A/Hong Kong/H090-751-V20/2009
A/Fuzhou/01/2009
A/Shanghai/37T/2009
A/Sichuan-Wenjiang/SWL456/2009
A/Hong Kong/H090-770-V10/2009
A/panda/Sichuan/01-GG/2009
A/giant panda/Ya'an/01/2009(H1N1)
A/swine/Hong Kong/158/1993
A/swine/Hong Kong/1733/2002
A/swine/Guangdong/05/2009
A/AA/Huston/1945
A/Hong Kong/1870/2008(H1N1)
A/Niigata/08F188/2009
A/Thailand/CU-H565/2009
0.02

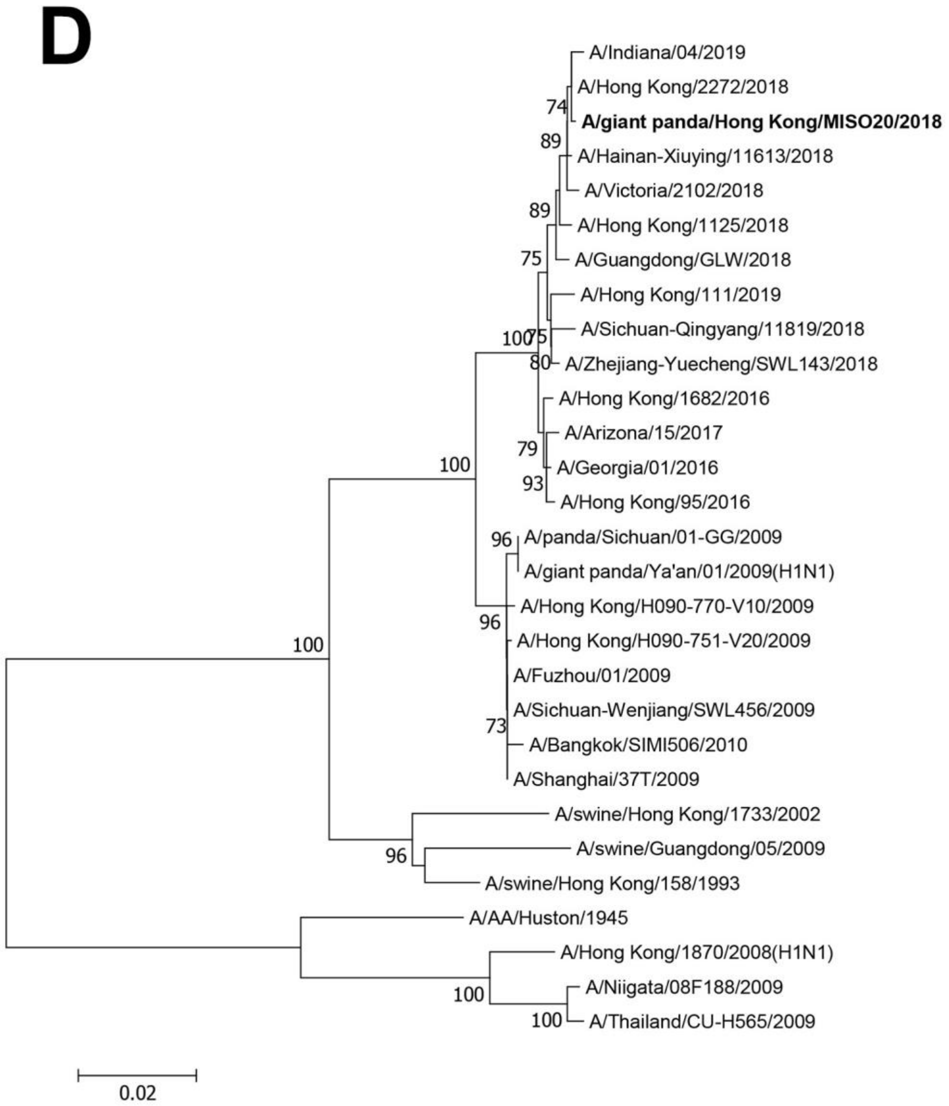
D
A/Indiana/04/2019
A/Hong Kong/2272/2018
A/giant panda/Hong Kong/MISO20/2018
A/Hainan-Xiuying/11613/2018
A/Victoria/2102/2018
A/Hong Kong/1125/2018
A/Guangdong/GLW/2018
A/Hong Kong/111/2019
A/Sichuan-Qingyang/11819/2018
A/Zhejiang-Yuecheng/SWL143/2018
A/Hong Kong/1682/2016
A/Arizona/15/2017
A/Georgia/01/2016
A/Hong Kong/95/2016
A/panda/Sichuan/01-GG/2009
A/giant panda/Ya'an/01/2009(H1N1)
A/Hong Kong/H090-770-V10/2009
A/Hong Kong/H090-751-V20/2009
A/Fuzhou/01/2009
A/Sichuan-Wenjiang/SWL456/2009
A/Bangkok/SIMI506/2010
A/Shanghai/37T/2009
A/swine/Hong Kong/1733/2002
A/swine/Guangdong/05/2009
A/swine/Hong Kong/158/1993
A/AA/Huston/1945
A/Hong Kong/1870/2008(H1N1)
A/Niigata/08F188/2009
A/Thailand/CU-H565/2009
74
89
89
75
100
75
80
79
93
100
96
96
100
73
96
100
100
0.02

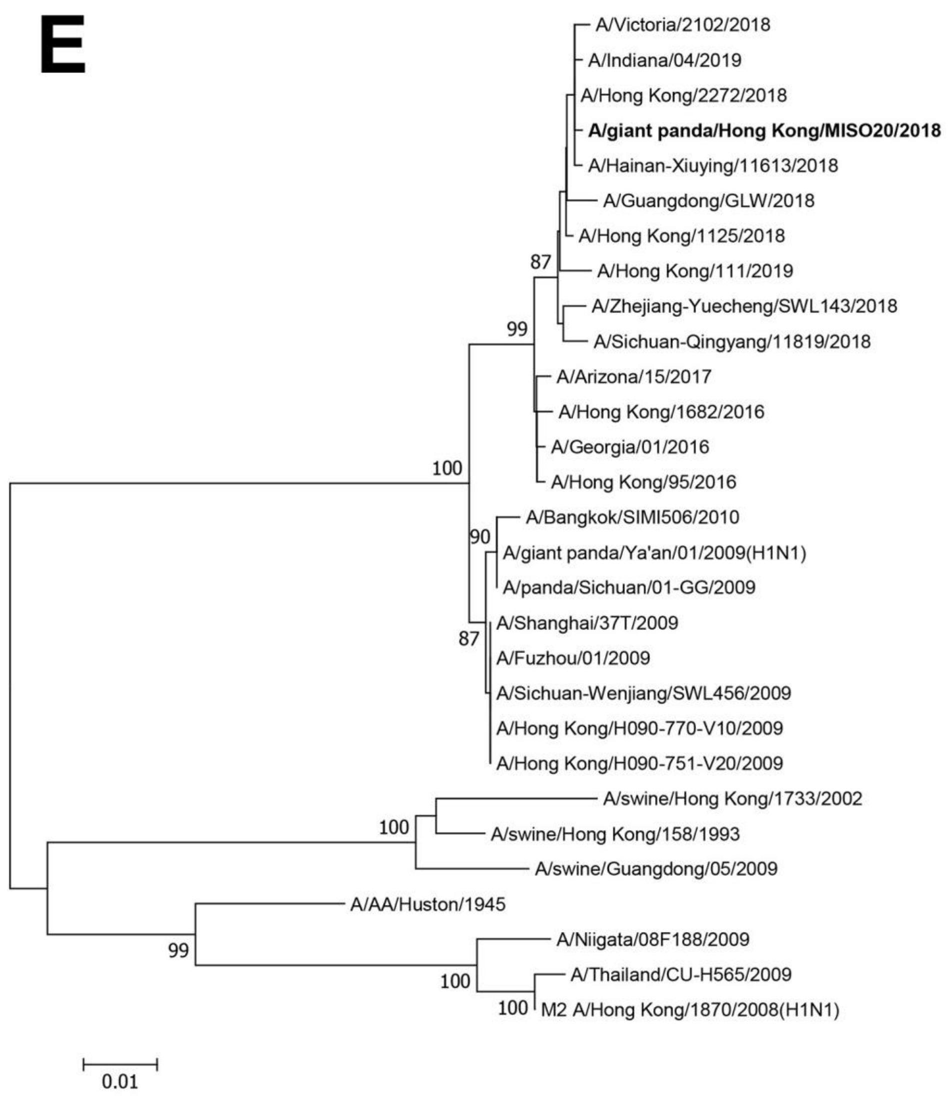
E
A/Victoria/2102/2018
A/Indiana/04/2019
A/Hong Kong/2272/2018
A/giant panda/Hong Kong/MISO20/2018
A/Hainan-Xiuying/11613/2018
A/Guangdong/GLW/2018
A/Hong Kong/1125/2018
87
A/Hong Kong/111/2019
A/Zhejiang-Yuecheng/SWL143/2018
99
A/Sichuan-Qingyang/11819/2018
A/Arizona/15/2017
A/Hong Kong/1682/2016
A/Georgia/01/2016
100
A/Hong Kong/95/2016
A/Bangkok/SIMI506/2010
90
A/giant panda/Ya'an/01/2009(H1N1)
A/panda/Sichuan/01-GG/2009
A/Shanghai/37T/2009
87
A/Fuzhou/01/2009
A/Sichuan-Wenjiang/SWL456/2009
A/Hong Kong/H090-770-V10/2009
A/Hong Kong/H090-751-V20/2009
A/swine/Hong Kong/1733/2002
100
A/swine/Hong Kong/158/1993
A/swine/Guangdong/05/2009
A/AA/Huston/1945
A/Niigata/08F188/2009
99
A/Thailand/CU-H565/2009
100
100
M2 A/Hong Kong/1870/2008(H1N1)
0.01

F

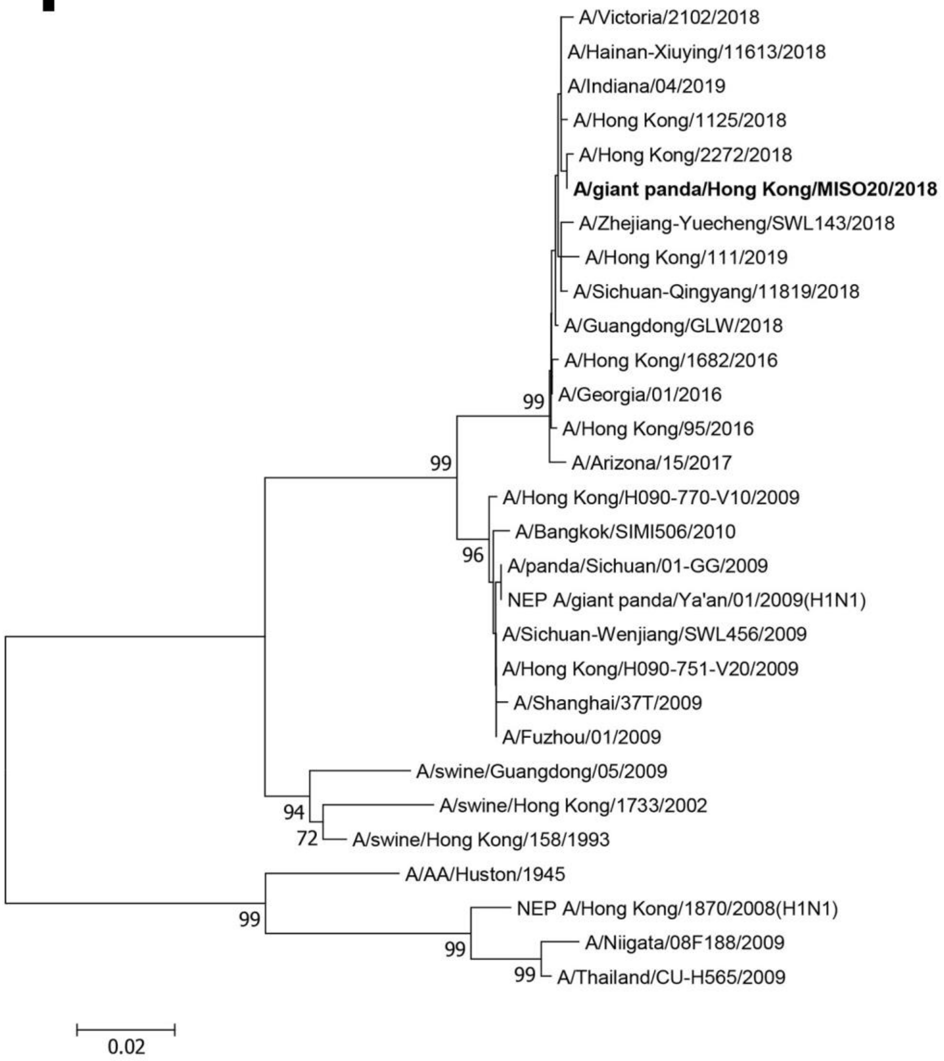

Appendix Figure. Phylogenetic analyses of 6 gene sequences of influenza A(H1N1)pdm09 (A/giant panda/Hong Kong/MISO20/2018) isolated from a giant panda and other previously characterized pH1N1. The trees were constructed by the neighbor-joining method using Kimura 2-parameter in MEGA6. The analyses included 2,238 nt positions in polymerase basic protein 2 (A), 2,262 in polymerase basic protein 1(B), 2,145 in polymerase acidic protein (C), 1,492 in nucleoprotein (D), 971 in matrix protein (E), and 824 in nonstructural protein gene sequences. Scale bars indicate nucleotide substitutions per site. Bootstrapping was performed with 1,000 replicates; only bootstrap values $\geq$700 are shown. Bold text indicates the giant panda isolate in this study.
